# Notch Signalling in the Hippocampus of Patients With Motor Neuron Disease

**DOI:** 10.3389/fnins.2019.00302

**Published:** 2019-04-05

**Authors:** Ulises Gómez-Pinedo, Lucía Galán, Jordi A. Matías-Guiu, Vanesa Pytel, Teresa Moreno, Antonio Guerrero-Sola, Jorge Matías-Guiu

**Affiliations:** ^1^Laboratory of Neurobiology, Institute of Neurosciences, IdISSC, Hospital Clínico San Carlos, Universidad Complutense de Madrid, Madrid, Spain; ^2^Department of Neurology, Institute of Neurosciences, IdISSC, Hospital Clínico San Carlos, Universidad Complutense de Madrid, Madrid, Spain

**Keywords:** ALS, Notch, NICD, APP, ADAM10, ADAM17, BACE1, hippocampal neurogenesis

## Abstract

**Introduction:**

The Notch signalling pathway regulates neuronal survival. It has some similarities with the APP signalling pathway, and competes with the latter for α- and γ-secretase proteolytic complexes. The objective of this study was to study the Notch signalling pathway in the hippocampi of patients with motor neuron disease.

**Methods:**

We studied biological material from the autopsies of 12 patients with motor neuron disease and 4 controls. We analysed the molecular markers of the Notch and APP signalling pathways, TDP43, tau, and markers of neurogenesis.

**Results and Conclusion:**

Low NICD expression suggests Notch signalling pathway inactivation in neurons. Inactivation of the pathway despite increased Notch1 expression is associated with a lack of α-secretase expression. We observed increased β-secretase expression associated with activation of the amyloid cascade of APP, leading to increases in amyloid-β and AICD peptides and decreased levels of Fe65. Inactivation of the Notch signalling pathway is an important factor in decreased neurogenic response in the hippocampi of patients with amyotrophic lateral sclerosis.

## Introduction

The Notch signalling pathway regulates cell migration and growth, synaptic plasticity, and neuronal survival (Ables et al., 2011). Notch proteins are highly conserved transmembrane receptors with such pleiotropic functions as neuronal development and organ homeostasis, and are activated by ligand binding. The ligand-receptor association triggers sequential proteolytic processes via α- and γ-secretases. Proteolysis generates a Notch intracellular domain (NICD) that may translocate to the nucleus. There is an evident parallel between the Notch and amyloid precursor protein (APP) signalling pathways, which compete for proteolytic complexes; an association between both pathways has therefore been suggested.

Notch1, the most extensively studied Notch receptor, is expressed in the cortex and hippocampus, and may be involved in neurodegeneration (Woo et al., 2009). Notch1-deficient mice display memory impairment (Costa et al., 2003). It has been suggested that Notch1 participates in olfactory function (Brai et al., 2014), which is impaired in patients with Alzheimer disease (AD) (Berezovska et al., 1998; Moehlmann et al., 2002; Brai et al., 2016) and in experimental models of familial AD secondary to presenilin mutations (Okochi et al., 2002). Amyotrophic lateral sclerosis (ALS) is a neurodegenerative disease affecting motor neurons in the brain, brainstem, and spinal cord (Rowland and Shneider, 2001). However, as recent anatomical pathology studies of ALS have shown that degeneration affects not only motor areas but also such other structures as the hippocampus (Coan and Mitchell, 2015), we aimed to determine whether the Notch pathway is active in the hippocampi of patients with ALS.

## Materials and Methods

We studied biological samples from the autopsies of 12 patients with ALS or ALS with frontotemporal dementia (ALS-FTD) who died between 2006 and 2017 and met diagnostic criteria for ALS (Ludolph et al., 2015). Ten patients died due to respiratory insufficiency during the terminal stage of ALS, one patient due to cardiac arrest attributed to bulbar involvement, and the remaining patient due to a concomitant cerebral haemorrhage. We also studied the brains of 4 controls, who died during hospitalisation due to non-neurological diseases and had no history of neurodegenerative disease. Only 10 brains were used for the study of neurogenesis. Patients or their families expressed in writing their consent for the brain to be donated for research. Autopsies were performed within 2–6 h after death, in accordance with our centre’s protocol and Spanish national regulations. The procedure, which we described in a previous article (Gómez-Pinedo et al., 2016), is outlined in Supplementary Material 1. Briefly, five slides were used per patient and IHC, analysing in each slides 32 fields (per hippocampal zone: CA1, CA2, CA3, and dentate gyrus). ImageJ version 1.46r was used when the unit of measurement was the amount of labelling per field (optical density [OD]). Inclusions were calculated as the number of stained inclusions observed in neurons divided by the mean number of neurons per field (percentage of cells per mm^2^). Statistical analysis was performed using the SPSS statistics software, version 20.0. GraphPad Prism version 5.0 was used to plot graphs and to calculate Pearson correlation coefficients between the parameters studied. Data are expressed as mean ± SD. Due to the small size of our sample, means were compared using the non-parametric Mann–Whitney U test. Statistical significance was set at *p* < 0.05.

## Results

### Notch1 Expression

**FIGURE 1:**
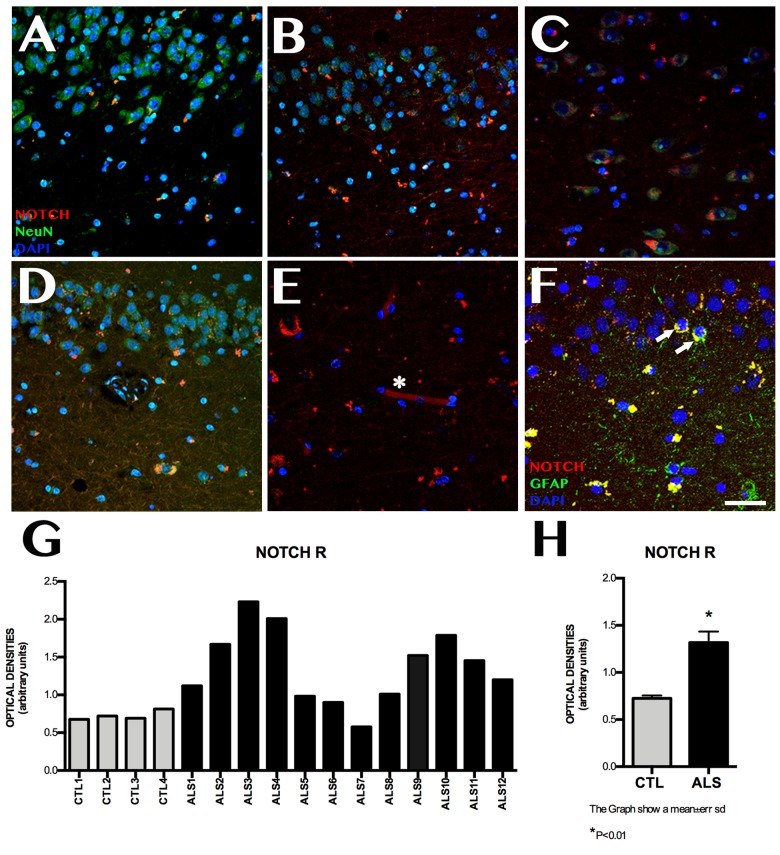
Notch1 expression. Confocal microscopy images from controls and patients with ALS, showing immunohistochemical expression of Notch1. Labelling is heterogeneous in several areas of the hippocampus **(A–D)**, and is more marked in CA1, in the cytoplasm of neurons **(A)**, and in the granular neurons of the dentate gyrus (**E**, asterisks). The subgranular zone shows labelling near capillaries (**E**, asterisk) and faint labelling in GFAP-positive cells from the subgranular zone (**F**, arrows). The graph in G shows quantitative data from the hippocampi (including CA1, CA2, CA3, CA4, and dentate gyrus) of all patients and controls. Values are heterogeneous among patients but generally higher in patients than in controls. The graph in H shows the mean values for patients and controls, which are significantly higher in patients (*p* < 0.05). Images correspond to the following areas: A, CA1 control; B, CA2 ALS; C, CA3 ALS; D, CA1 and subgranular zone ALS; E, CA3; and F, subgranular zone ALS. Scale bar: 50 μm. Graphs express data as means **(G)** and standard deviation **(H)**.

Notch1 expression was observed at higher levels in patients than in controls (Figure 1). Mean labelling per field was 1.315 ± 0.448 OD in patients and 0.725 ± 0.061 OD in controls (*p* < 0.02). Figure 1 also shows differences between patients: one patient with ALS-FTD showed lower Notch1 expression than controls, 2 patients with ALS showed similar expression to that observed in controls, and the remaining patients displayed clearly greater Notch1 expression. Patients and controls showed different labelling patterns. In controls, labelling was mainly observed in the cytoplasm and dendrites in CA1, especially in the polymorphic layer of the subgranular zone (SGZ). In patients, however, labelling was heterogeneous in all hippocampal areas, being more evident in granular neurons and remarkable in CA1, CA3, the polymorphic layer of CA4, and the SGZ; Notch1 expression was observed in neurons and to a lesser extent in astrocytes and neuronal processes, following a slight synaptic pattern in CA1 and in capillary walls (Figure 1).

### NICD Expression

**FIGURE 2:**
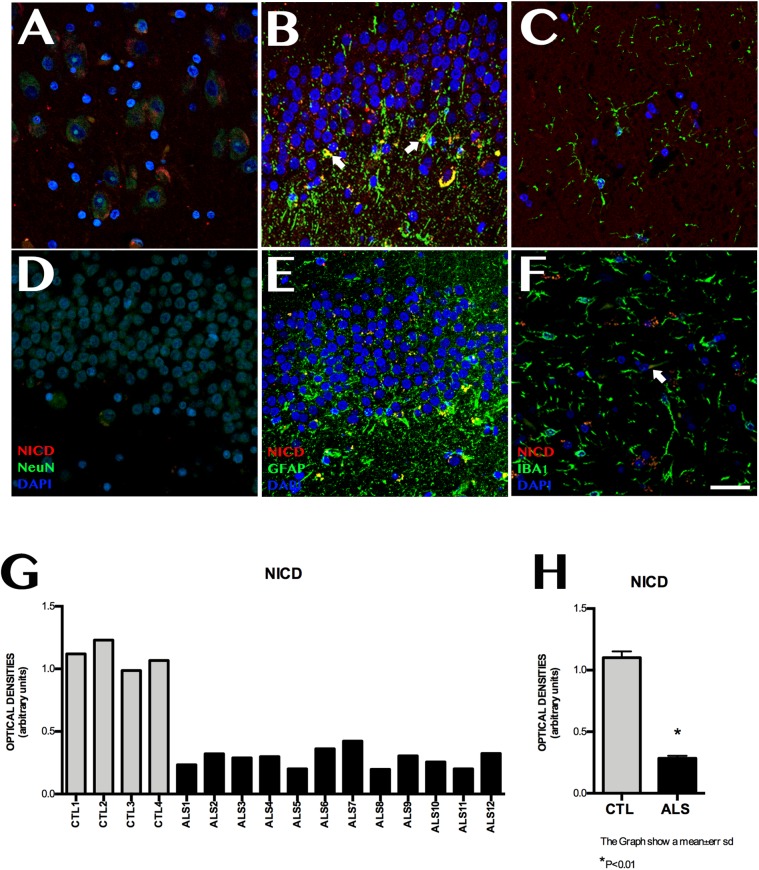
NICD expression in the hippocampi of patients with ALS and controls. Immunohistochemical images of NICD expression obtained by confocal microscopy, showing clear differences in labelling patterns between patients and controls. The most significant differences were observed in the granular layer and the subgranular zone. Controls showed more marked labelling than patients with ALS **(A–C)**, especially in the granular zone, Controls displayed cells (astrocytes) coexpressing GFAP and NICD in the subgranular zone (**B** arrows), whereas patients showed faint labelling in the granular layer **(D,E)**, in addition to Iba1-positive cells (**F**, arrows). The graph in **F** shows quantitative data from the hippocampi (including CA1, CA2, CA3, CA4, and dentate gyrus) of all patients and controls. Values are higher in controls than in patients. The graph in **G** shows significant differences in mean values between patients and controls, with controls showing higher expression (*p* < 0.05). Images **A**, CA3; **B**, **D**, and **E** show the granular layer and subgranular zone, whereas image **C** and **F** shows the CA2. Scale bar: 50 μm. Graphs express data as means **(G)** and standard deviation **(H)**.

NICD expression was variable in patients with ALS, and was observed at lower levels than in controls (Figure 2). Mean labelling per field was 0.228 ± 0.070 OD in patients and 1.101 ± 0.101 OD in controls (*p* < 0.0001). NICD expression is inversely related to Notch1 expression and shows homogeneous values, since it behaves similarly in all patients with ALS, including those with ALS-FTD; NICD labelling in patients was lower than that observed in controls. In patients with ALS, NICD labelling was mainly observed in cell nuclei, especially in granular neurons, neurons near the SGZ, and in the polymorphic layer. The SGZ contained astrocytic cells coexpressing GFAP and NICD, especially in patients with greater numbers of extracellular amyloid plaques (Supplementary Material 2). Patients with no plaques or isolated plaques showed no NICD labelling, except in isolated astrocytes near the SGZ (Supplementary Material 2). In controls, NICD was mainly expressed in neurons of the SGZ and colocalised with GFAP. Labelling was also observed in microglia (Iba1-positive cells) (Figure 2).

### ADAM10, ADAM17, and BACE1 Expression

**FIGURE 3:**
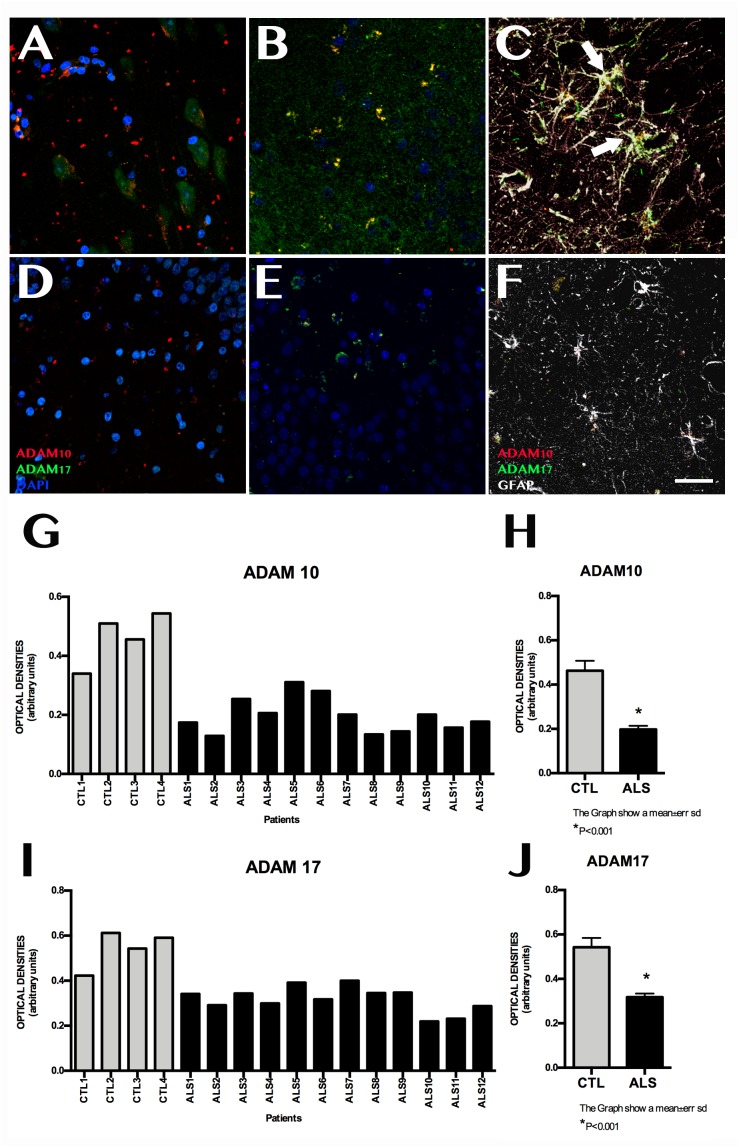
ADAM10 and ADAM17 expression in the hippocampus. Images of the hippocampus obtained using confocal microscopy after immunohistochemistry against ADAM10 and ADAM17 show lower marker expression in controls **(A–C)** than in patients **(D–F)**. Labelling was mainly observed in the granular layer of the dentate gyrus; the subgranular zone showed weak, diffuse labelling **(D–F)**. Labelling could not be observed in the remaining areas of the hippocampus in some patients. Controls displayed denser labelling, which was observed in the granular layer and subgranular zone, where astrocytes coexpressed ADAM10 and ADAM17 (C, arrows). Quantitative data for ADAM10 and ADAM17 are shown in graphs **(G)** and **(H)**, respectively. Graphs show similar ADAM10 and ADAM17 expression in both patients and controls; this is further confirmed by graphs **(I)** and **(J)**, which show statistically significant differences (*p* < 0.05). Images **A** and **B** correspond to representative areas of the dentate gyrus and the subgranular zone in controls, whereas images **C** and **D** display the same areas in patients. Scale bar: 25 μm. Graphs **E** and **F** express data as means, and graphs **G** and **H** as means ± standard deviation.

**FIGURE 4:**
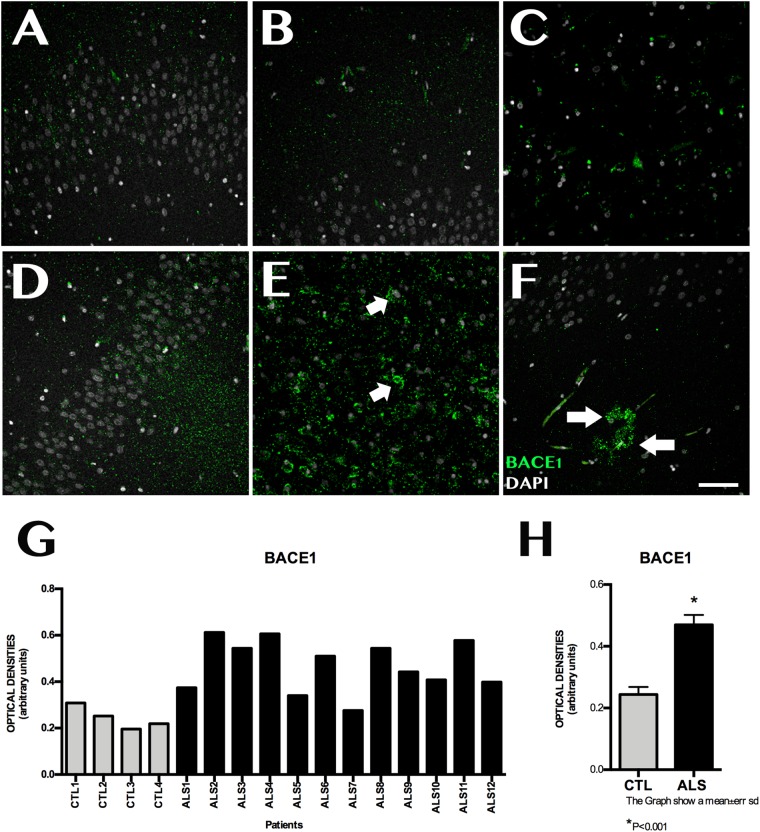
BACE1 expression in the hippocampus. **(A–C,F)** displays the dentate gyrus/subgranular zone and CA1 in controls **(D–F)** shows the increased BACE1 expression in patients, located in the subgranular zone (**E**, arrow) and in areas with amyloid plaques (**F**, arrows). Increased BACE1 expression is observed in nine patients; the remaining three showed similar levels to those observed in controls **(G)**. The graph in **(H)** expresses data as means, and shows significant differences (*p* < 0.05). Image **G** expresses data as means and graph **H** as means ± standard deviation. Scale bar: 50 μm.

ADAM10 expression was 0.199 ± 0.060 OD in patients and 0.462 ± 0.089 OD in controls (*p* = 0.0015); it was lower in all patients with ALS (Figure 3). ADAM17 expression was 0.230 ± 0.057 OD in patients and 0.542 ± 0.084 OD in controls (*p* = 0.0015); again, expression was lower in all patients than in controls (Figure 3). In patients with ALS, ADAM10 and ADAM17 expression was similar in terms of location, although ADAM10 expression was slightly more marked in the granular layer and less marked in the SGZ. ADAM17 expression was more diffuse, whereas ADAM10 expression was more defined and homogeneous (Supplementary Material 4). In controls, expression of both ADAM10 and ADAM17 was more marked in the SGZ (Supplementary Material 3), colocalised with GFAP, and was also observed in neurons. BACE1 expression was evaluated with OD and by determining the number of cells labelled by the antibody. BACE1 expression was 0.475 ± 0.114 OD in patients with ALS and 0.243 ± 0.048 OD in controls (*p* < 0.0001). Higher levels were observed in the majority of patients with ALS, however, three patients showed similar levels to those of controls (Figure 4). A total of 13.73 ± 2.97 cells per field in patients with ALS and 8.00 ± 2.588 in controls were labelled by anti-BACE1 antibody (*p* = 0.0047). Similarly, the antibody labelled more cells in all patients with ALS, with the exception of the 3 patients showing similar BACE1 expression to those of controls as measured by OD (Figure 4).

### APP, Amyloid-β, AICD, and Fe65 Expression

APP expression was 5689 ± 2036 OD in patients and 2544 ± 1209 OD in controls (*p* = 0.0076). APP intracellular domain (AICD) expression was elevated in 5 patients, whereas the remaining 5 showed similar levels to those observed in controls (Supplementary Material 4). Amyloid-β (Aβ) expression was elevated in all patients with ALS (38 249 ± 24 449 OD vs. 6003 ± 2231 OD in controls; *p* = 0.0127). AICD expression was 14.10 ± 3.07 OD in patients and 9.2 ± 3.49 OD in controls (*p* = 0.0153). In patients displaying Aβ expression, AICD was observed in astrocytes and microglia surrounding amyloid plaques; it was also expressed in dendrites. Elevated AICD expression was observed in all patients with ALS, with the exception of 3 who showed similar levels to those observed in controls (Supplementary Material 4). Fe65 expression was 13.90 ± 3.24 OD in patients and 22.60 ± 4.03 OD in controls (*p* = 0.0006). Decreased Fe65 expression was observed in all patients, with the exception of 3 who showed similar expression to that of controls; these were not the same patients showing increased AICD expression (Supplementary Material 4). Ten patients displayed amyloid plaques with varying degrees of labelling. Plaques were frequently diffuse and isolated; only 4 of these patients showed prominent plaques, mainly located in CA1, CA3, and cortical regions.

### Expression of Markers of Neurogenesis

Shows data for 10 of the 12 patients with ALS (the remaining 2 patients were not included in this part of the study for technical reasons). Patients showed a marked decrease in proliferative and neurogenic activity in the hippocampus. Proliferation in the SGZ was analysed using the proteins PCNA and Ki67, expressed during cell division. The number of cells labelled with these 2 markers was significantly lower in patients than in controls: 0.47 ± 0.72 cells/μm^2^ vs. 2 ± 1.41 cells/μm^2^ for PCNA labelling, and 0.29 ± 0.72 cells/μm^2^ vs. 1.65 ± 0.96 cells/μm^2^ for Ki67 labelling (*p* < 0.01). The study of such immunohistochemical markers of human pluripotent cells as GFAPδ in the dentate gyrus identified fewer labelled cells in patients than in controls: 1.18 ± 0.83 cells/μm^2^ vs. 5.7 ± 1.81 cells/μm^2^ (*p* < 0.01). The markers DCX, TuJ1, and PSA-NCAM were used to study neuroblast expression in the dentate gyrus. PSA-NCAM expression was reduced in patients with ALS (0.39 ± 0.43 cells/μm^2^ vs. 7.3 ± 4.29 cells/μm^2^; *p* < 0.01). The other 2 markers could not be viewed since they only labelled small dendritic projections but no somata.

### TDP43 and Tau Expression

Cytoplasmic TDP43 expression was evaluated using staining for phosphorylated TDP43; expression was determined at 55.75 ± 5.97 OD in patients vs. 58.8 ± 5.167 OD in controls; differences were not statistically significant. However, the number of TDP43-positive cytoplasmic inclusions per field did show statistically significant differences between patients and controls (20.30 ± 10.32 vs. 1.504 ± 0.993; *p* = 0.0004). All patients displayed TDP43-positive cytoplasmic inclusions. Quantification of phosphorylated tau (phospho-tau) expression revealed levels of 26 106 ± 20 413 OD in patients and 4824 ± 2896 OD in controls (*p* = 0.0395). Only 6 patients showed increased phospho-tau expression; we observed neurofibrillary tangles mainly in CA3 and CA1, and staining of small fibres in CA1 axonal projections. The remaining patients showed similar levels of phospho-tau expression to that of controls.

### Correlations Between Molecular Markers

Supplementary Material 5 show the correlations between the various molecular markers. In the Supplementary Tables 1, 2 describe the correlations between molecular markers of NOTCH pathway and the correlation with NOTCH and adult neurogenesis.

### Clinical Correlations

Patients (7 men and 5 women) were aged between 37 and 87 years at symptom onset; onset was spinal in 5 cases and bulbar in 7. Survival times ranged from 4 months to 14 years. Two patients also had dementia; one case was attributed to FTD due to the associated aphasia. Seven patients underwent a genetic study. Three patients showed a *SOD1* mutation, which was pathogenic in only one case. One patient had a pathogenic mutation of the gene coding for TDP43. No patient had more than 20 repeats in *C9ORF72*. No correlations were found between molecular data and such clinical characteristics as age, sex, form of onset, survival time, or presence of any of the genetic variants detected (Supplementary Table 3).

## Discussion

### Notch1 Is Overexpressed, Whereas NICD Is Underexpressed

**FIGURE 5:**
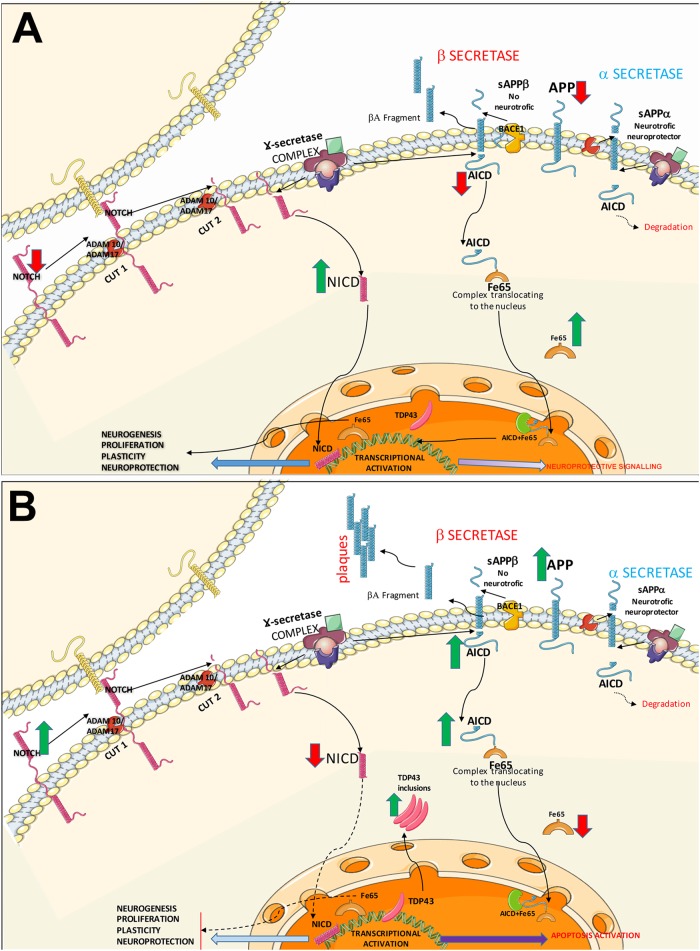
Diagram of the Notch and APP signalling pathways. The diagram shows the possible common mechanisms between both pathways in controls **(A)** and patients with ALS **(B)**. **(A)** Healthy control. In the Notch signalling pathway, Notch1 activation requires direct contact between the membranes of 2 cells. Once the receptor is active, the protein undergoes two consecutive cleavages by α-secretases (ADAM10 and ADAM17), followed by transmembrane cleavage by γ-secretases. NICD is internalised into the nucleus, activating transcription factors and promoting mechanisms of cell plasticity, proliferation, and neurogenesis. The APP signalling pathway may follow two routes. The non-amyloidogenic (neuroprotective) APP pathway: after α-secretase–mediated cleavage, γ-secretases generate AICD and decrease APP levels. Cytosolic adaptor protein Fe65 binds to AICD; this complex translocates to the nucleus where it interacts activating signalling to promotes neurite growth and cell plasticity. The second pathway is the amyloidogenic APP pathway, where APP is processed by α-secretase and γ-secretase, forming Aβ. AICD is also generated, which may activate NICD expression via the lysosomal system or interact with Fe65. Both pathways converge in the activation of substrates by α-secretases. **(B)** A possible pathogenic mechanism in ALS is increased Notch1 expression due to poor processing of α-secretases, given the competition between the Notch and APP pathways. This results in poor NICD activation, leading to transcription block and the subsequent decrease in plasticity, proliferation, and neurogenesis. In the APP pathway, the competition for α-secretases (ADAM10, ADAM17) causes β-secretase activation, leading to the formation of Aβ oligomers; Aβ plaque formation in the intracellular space generates AICD, directly affecting Fe65 expression. AICD and Fe65 form a complex that translocates to the nucleus, where it may activate the apoptotic pathway. Increased AICD expression and low Fe65 activity may affect other lysosomal degradation pathways, increasing cytoplasmic TDP43 inclusions.

Notch1 is generated by a convertase enzyme in the Golgi apparatus via S1 cleavage and subsequently transported to the cell membrane, where it is expressed as a heterodimeric transmembrane protein (Lieber et al., 2002). Notch1 overexpression has previously been observed in other neurodegenerative diseases (Nagarsheth et al., 2006; Lathia et al., 2008) activation of the Notch signalling pathway has been found to play a role in ageing and memory (Ables et al., 2011; Alberi et al., 2013). Notch1’s role in ALS has previously been observed in several experimental studies, which report conflicting findings. Wang et al. (2015) observed that the Notch signalling pathway is activated *in vitro* models and SOD1^G93A^ mice, and that Notch suppression with a Notch1 signalling inhibitor significantly reduced neuronal apoptosis. Nonneman et al. (2018), in contrast, studied Notch signalling in the spinal cords of SOD1^G93A^ mice and patients with sporadic ALS, finding increased pathway activation in reactive GFAP-positive astrocytes. Astrocyte-specific inactivation of Jagged-1 in presymptomatic SOD1^G93A^ mice increased the activation of the Notch signalling pathway and accelerated disease progression without affecting disease onset. In a study of *Drosophila*, Yang et al. (2015) observed that dipeptide repeat proteins associated with a repeat expansion in *C9ORF72*, present in patients with FTD and ALS, were accompanied by Notch signalling suppression. We did not observe Notch signalling pathway activation in the hippocampi of our patients: while they displayed increased Notch1 expression, NICD expression was significantly decreased. Our results are consistent with those reported by Ma et al. (2017), who observed decreased NICD signalling in spinal cord motor neurons of SOD mice; this decrease was correlated with disease expression. Disease onset occurred between 90 and 120 days of age, at which time NICD levels progressively decreased in motor neurons. After binding to one of its transmembrane ligands, Notch undergoes sequential cleavage from NICD first by α-secretase and then by γ-secretase; NICD is then internalised into the nucleus (Figure 5). Our results suggest that although Notch expression increases, probably in response to increased expression of its ligands (Nonneman et al., 2018). This is consistent with the findings of Ma et al. (2017), who suggest the involvement of some mechanism within the cell that interrupts activation of the pathway. However, our patients showed NICD expression in astrocytes (GFAP-positive cells) and microglia (Iba1-positive cells), suggesting Notch pathway activation in these cells. This observation coincides with the results of Nonneman et al. (2018), who report Notch signalling pathway inactivation in neurons and activation in glial cells. This is consistent with the hypothesis of neuronal loss and gliosis in ALS (Rowland and Shneider, 2001).

### APP and Notch1 Expression

In a previous study, we observed amyloid cascade activation in the hippocampi of patients with ALS-FTD, in the form of increased APP and Aβ expression. AICD expression was variable, as the protein was not overexpressed in all patients; we also observed decreased Fe65 expression, suggesting that AICD may have bound to Fe65 and been internalised into the nucleus, as occurs when the APP signalling pathway is activated (Gómez-Pinedo et al., 2016). These results are consistent with our findings from an *in vivo* study of patients with ALS using PET with amyloid tracers (Matías-Guiu et al., 2016). The fact that Notch and APP signalling pathways compete with each other for α- and γ-secretase underscores the need to analyse the link between these pathways. In order to generate AICD, APP undergoes sequential cleavage, first by α- or β-secretase and subsequently by γ-secretase. When cleavage is first mediated by β-secretase, APP generates Aβ peptides, which are oligomerised in the form of aggregates. APP cleavage by γ-secretase generates AICD (Figure 5). In this study, we observed increased APP and Aβ expression. Similarly, a rat model of AD showed that soluble Aβ_1-42_ suppresses Notch1 and NICD expression (Zhang et al., 2016). AICD generated by γ-secretase–mediated cleavage is rapidly degraded by the endosomal-lysosomal system. Fe65 stabilises AICD; together, both molecules localise to the nuclear compartment, where they bind the histone acetylase Tip60, forming AFT complexes (Von Rotz et al., 2004). AICD also competes with NICD within the nucleus. A study of human embryonic kidney cells found colocalisation in AFT complexes; NICD can localise to the nucleus together with Fe65 and Tip60 in the absence of AICD (Konietzko et al., 2010).

### Fe65 Expression Increases With Notch1 Overexpression

Fe65 binds to AICD; in the nucleus, this complex is involved in regulating the transcription of certain genes, including the gene coding for APP (Cao and Südhof, 2001); *in vitro* studies have shown colocalisation of AICD and TDP43 in the nucleus (Wang et al., 2014). Fe65 is highly expressed in the hippocampus (Kesavapany et al., 2002). Fischer et al. (2005) observed increased Notch1 expression, interaction between APP and Notch1, and NICD binding to Fe65 in the cerebral cortex of adults with Down syndrome, which is associated with enhanced APP production. NICD may colocalise with AICD, Fe65, and Tip60, interrupting the formation of the AFT complex and playing a protective role by inhibiting AFT complex–induced cell death (Kim et al., 2007). Our study shows that decreased Fe65 expression coincides with decreased NICD expression and increased Notch1 expression; this is consistent with the results of a previous study by our research group (Gómez-Pinedo et al., 2016).

### Notch Signalling Pathway Inactivation May Explain Decreased Neurogenic Response in the Hippocampus

The hippocampus, one of the classic neurogenic niches of the adult brain, constantly generates neurons throughout life (Palmer et al., 1997; Eriksson et al., 1998; Seri et al., 2004). Granule cells are born in the SGZ of the dentate gyrus; they migrate to the granular layer and integrate into the neural network. However, these cells are estimated to be less numerous than those born in the subventricular zone. AD (Hollands et al., 2016) and other neurodegenerative diseases (Winner and Winkler, 2015) have been found to be associated with alterations in hippocampal neurogenesis. Our research group has described decreased hippocampal neurogenesis in patients with ALS, which stands in contrast with the increased neurogenesis observed in the subventricular zone in these patients (Galán et al., 2017). The Notch signalling pathway has been linked to hippocampal neurogenesis as the Notch receptor is expressed in neural stem cells (Traiffort and Ferent, 2015). It has been suggested that this pathway may alter the number of neural stem cells by acting on cell survival (Ables et al., 2010), self-renewal (Aguirre et al., 2010), and differentiation (Breunig et al., 2007), and may act as a mechanism of communication between a neural stem cell and its descendants (Semerci et al., 2017). Our results show that expression of markers of proliferation (Ki67), differentiating cells (GFAPδ), and differentiation (PSA-NCAM) is significantly correlated with NICD expression and negatively correlated with Notch1, which suggests that decreased neurogenesis in ALS (Galán et al., 2017) may be associated with Notch signalling pathway inactivation. Interestingly, some drugs that increase Notch signalling have been found to promote hippocampal neurogenesis (Xue et al., 2017).

### Expression of α- and β-Secretases

The role of α- and β-secretases comes to the forefront in view of 3 main findings: Notch signalling inactivation despite increased Notch1 expression; decreased hippocampal neurogenesis resulting from Notch signalling inactivation; and APP signalling pathway activation (Gómez-Pinedo et al., 2016). α-Secretase acts on Notch during S2 cleavage in response to ligand binding, which induces a conformational change. The cleaved form that remains bound to the membrane is called NEXT and will be the substrate for γ-secretase–mediated proteolysis (S3 cleavage), whereas the resulting intracellular Notch fragments are short-lived. α-Secretases are membrane-anchored, zinc-dependent members of the A disintegrin and metalloproteinase (ADAM) family. ADAM includes a wide range of proteins with protease and adhesive domains, which play a key role in cell-cell and cell-matrix interactions in various important biological cell processes. The role of α-secretase involves ADAM10 and ADAM17, and to a lesser extent ADAM12 and ADAM9. Little information is available on ADAM molecular expression in ALS. Our study detected decreased ADAM10 and ADAM17 expression, which may explain S2 cleavage inhibition (Groot and Vooijs, 2012). Our results do not allow us to determine the reason for reduced α-secretase expression, although the discovery of some factors involved in ADAM10 regulation permits us to propose several hypotheses. Synapse-associated protein 97 binds to ADAM10, creating a complex that enables its transportation from the endoplasmic reticulum to the cell membrane. Glucagon-like peptide-1 is associated with decreased ADAM10 expression and lower Aβ levels (Saftig and Lichtenthaler, 2015). The role of the TspanC8 subgroup of tetraspanins (including Tspan5, Tspan10, Tspan14, Tspan15, Tspan17, and Tspan33) is especially noteworthy (Dornier et al., 2012; Haining et al., 2012). TspanC8 tetraspanins interact with ADAM10 and regulate the cleavage of ADAM10 substrates. Tspan5, Tspan10, and Tspan14 regulate ADAM10-dependent Notch signalling (Zhou et al., 2014); Tspan15 promotes ADAM10-mediated N-cadherin cleavage; and Tspan14 reduces GPVI cleavage (Noy et al., 2016); changes in tetraspanin expression may alter the action of ADAM10. Another hypothesis suggests competition with other substrates where ADAM10 is also involved, such as the TNFα signalling pathway, which has been observed to be active in ALS (Tortarolo et al., 2017) and cause cell alterations (Olmos and Lladó, 2014); this is probably related to microglial activation, one of the first events to be observed in experimental models of the disease (Beers et al., 2011; Liao et al., 2012; Liu and Wang, 2017; Gómez-Pinedo et al., 2018). The observation of NICD expression in Iba1-positive cells in our patients supports this hypothesis. NICD expression is directly correlated with ADAM10 and ADAM17 expression, whereas Notch1 expression decreases with increased metalloprotease expression; this supports our hypothesis that decreased Notch1 and NICD expression is associated with Notch signalling pathway inactivation. Conversely, we observed increased β-secretase expression (BACE1) (Yan et al., 2001); this was not unexpected given the results of our previous study, which showed amyloidogenic APP pathway activation (Gómez-Pinedo et al., 2016), and other results from experimental studies into the interaction between BACE1 and TDP43 (Herman et al., 2012). It has been suggested that BACE1 may play a role in the Notch signalling pathway since it seems to be involved in maintaining the balance between hippocampal astrogenesis and neurogenesis in mice by regulating the Jag1-Notch pathway (Hu et al., 2013; He et al., 2014). In our study, BACE1 expression was significantly correlated with Notch1 expression and negatively correlated with NICD expression, which supports the hypothesis that the amyloidogenic APP pathway is activated in the hippocampi of patients with ALS, whereas the Notch signalling pathway is not.

### Lack of Correlation Between Notch Expression and Cytoplasmic TDP43 and Tau Expression

A previous study by our research group found no correlation between cytoplasmic TDP43 and APP; furthermore, the 2 proteins did not colocalise, although we did find a correlation between TDP43 expression and Aβ expression (Gómez-Pinedo et al., 2016). Zhan et al. (2013) report that TDP-43 upregulates a wide range of Notch target genes, leading to the activation of this cell differentiation pathway *in vivo*. Notch signalling pathway activation has been linked to prion disease (Ishikura et al., 2005), suggesting a common mechanism with ALS, considering that the C-terminal domain of TDP-43 has prion-like characteristics (Zhan et al., 2013). In our study, no correlation was observed between cytoplasmic TDP43 expression and Notch1 and NICD expression in patients; this contradicts the hypothesis that changes in the signalling pathway may be affected by TDP43. Our previous study detected tau overexpression (Gómez-Pinedo et al., 2016); we hypothesised that this finding may be linked to increased AICD expression resulting from APP pathway activation (Kim et al., 2003; Ghosal et al., 2009). Tau overexpression has also been reported by other researchers (Vintilescu et al., 2016), and the protein’s potential role in ALS has recently been reviewed (Moszczynski et al., 2018). Our study found no correlation with Notch, however.

### Study Limitations

Our study has a number of limitations, some of which were discussed in our previous article (Gómez-Pinedo et al., 2016). Autopsies were performed 2 to 6 h after death; this time interval is longer than those used in animal studies. Although different tissue samples were used, some of the patients included in this study were included in the previous studies. However, our sample also included new patients, reducing clinical bias: the two previous studies included a large proportion of patients with bulbar-onset ALS, with very short survival times, whereas this study mostly includes patients with spinal-onset ALS, some of whom had long survival times.

## Conclusion

To our knowledge, this is the first study to analyse the Notch signalling pathway in biological samples taken in tissue samples from the hippocampus from patients with ALS; In a recent paper, Nonneman et al. (2018) found that Notch signalling pathway is activated in the reactive astrocytes in the spinal cord of SOD1hG93A mice, as well as in patients with sporadic ALS and their finding of a upregulation of astrocytic Jagged-1 is in concordance with the our finding of the greater expression of Notch1 in our cases. Given the involvement of the Notch signalling pathway in cell survival, the finding that the pathway is not active in neurons (decreased NICD expression) is consistent with neuronal loss in patients with ALS. Inactivation of this pathway in neurons despite increased Notch1 expression is associated with a lack of α-secretase expression, preventing APP non-amyloidogenic signalling pathway activation; this was described in one of our previous studies. We also observed increased β-secretase expression in association with activation of the amyloid cascade of APP, leading to increased Aβ and AICD expression and decreased Fe65 expression. Inactivation of the Notch signalling pathway is an important factor in neuronal death in ALS and also plays a major role in decreased hippocampal neurogenic response in these patients, although the blockade of expression of Notch1 in oligodendrocyte precursor cells does not influence the evolution of the mutant SOD1hG93A mice (Eykens et al., 2018). Our study contributes to the understanding of molecular changes in cells due to ALS. Lack of α-secretase expression should be analysed in future studies, given its potential therapeutic implications.

## Ethics Statement

All participants or their relatives gave informed consent prior to inclusion in the study (including consent to autopsy). Autopsies were performed according to the procedures established by our hospital’s anatomical pathology department. This study was approved by the Clinical Research Ethics Committee of Hospital Clínico San Carlos. Data were managed in accordance with Spanish data protection legislation (Organic Law 15/1999 of 13 December). At the time the study was approved, no consent was necessary for publishing the results.

## Author Contributions

UG-P and LG contributed manuscript drafting, study concept and design, data acquisition, data analysis and interpretation, statistical analysis. JAM-G contributed manuscript drafting, study concept and design, data analysis and interpretation, statistical analysis. VP performed data analysis and interpretation, statistical analysis. AG-S performed data analysis, critical review of the manuscript. TM data acquisition, critical review of the manuscript. JM-G performed the study concept and design, data analysis and interpretation, study supervision, and manuscript drafting. All authors have read and approved the final version of the manuscript.

## Conflict of Interest Statement

The authors declare that the research was conducted in the absence of any commercial or financial relationships that could be construed as a potential conflict of interest.
