# Supplementary material for: Notch Signalling in the Hippocampus of Patients With Motor Neuron Disease
Source: Front Neurosci. 2019 Apr 5;13:302. doi: 10.3389/fnins.2019.00302 (PMC6460507; doi:10.3389/fnins.2019.00302)
Supplement: Supplementary file 1 [file Table_1.docx]

*Supplementary Table 1*

| **Correlation between molecular markers NOTCH pathway** | | | | |
| --- | --- | --- | --- | --- |
|  |  | ***r*** | **95% Confidence Interval** | **Correlation** |
| **NOTCH1 vs** | NICD | -0.5878 | -0.8602 to 0.0542 | Weak Negative Correlation |
|  | Fe65 | -0.5296 | -0.8463 to -0.0637 | Weak Negative Correlation |
|  | ADAM10 | -0.5443 | -0.8521 to -0.0432 | Weak Negative Correlation |
|  | ADAM17 | -0.6485 | -0.8909 to -0.1187 | Weak Negative Correlation |
|  | APP | 0.3769 | -0.2516 to 0.7818 | No Correlation |
|  | TDP43 | 0.1887 | -0.4321 to 0.6882 | No Correlation |
|  | Aβ | 0.7825 | 0.3788 to 0.9361 | Strong Correlation |
|  | BACE | 0.7994 | 0.4165 to 0.9414 | Strong Correlation |
|  | pTAU | 0.6782 | 0.1706 to 0.9013 | Strong Correlation |
|  |  |  |  |  |
| **NICD vs** | APP | -0.7192 | -0.9153 to -0.2472 | Negative Correlation |
|  | BACE | -0.7210 | -0.9159 to -0.2508 | Negative Correlation |
|  | Aβ | -0.5442 | -0.8520 to -0.4337 | Weak Negative Correlation |
|  | TDP43 | -0.5958 | -0.8717 to -0.0331 | Weak Negative Correlation |
|  | pTAU | -0.4866 | -0.8291 to 0.1212 | Weak Negative Correlation |
|  | Fe65 | 0.7497 | 0.3084 to 0.9254 | Strong Correlation |
|  | ADAM10 | 0.9021 | 0.6803 to 0.9725 | Strong Correlation |
|  | ADAM17 | 0.9446 | 0.8096 to 0.9847 | Strong Correlation |
|  |  |  |  |  |
| **Markers of neurogenesis** | | |  |  |
| **NOTCH1vs** | Ki67 | -0.5609 | -0.8585 to -0.01927 | Negative Correlation |
|  | GFAPδ | -0.5377 | -0.8495 to -005250 | Negative Correlation |
|  | PSA-NCAM | -0.5769 | -0.8646 to -0.004265 | Negative Correlation |
|  | GFAPα | 0.7210 | 0.2507 to 0.9159 | Strong Correlation |
|  |  |  |  |  |
| **NICD vs** | Ki67 | 0.8218 | 0.4690 to 0.9484 | Strong Correlation |
|  | GFAPδ | 0.8355 | 0.5026 to 0.9527 | Strong Correlation |
|  | PSA-NCAM | 0.8799 | 0.6182 to 0.9660 | Strong Correlation |
|  | GFAPα | -0.8318 | -0.9515 to -0.4933 | Negative Correlation |
