# Supplementary material for: Notch Signalling in the Hippocampus of Patients With Motor Neuron Disease
Source: Front Neurosci. 2019 Apr 5;13:302. doi: 10.3389/fnins.2019.00302 (PMC6460507; doi:10.3389/fnins.2019.00302)
Supplement: Supplementary file 2 [file Table_2.docx]

*Supplementary Table 2*

| **Correlation between molecular markers NOTCH pathway in CONTROLS** | | | | |
| --- | --- | --- | --- | --- |
|  |  | ***r*** | **95% Confidence Interval** |  |
| **NOTCH1 vs** |  |  |  |  |
|  | Fe65 | -0,9025 | -0,9980 to 0,4424 |  |
|  | ADAM10 | 0,8042 | -0,6911 to 0,9957 |  |
|  | ADAM17 | 0,6326 | -0,8381 to 0,9911 |  |
|  | APP | -0,2013 | -0,9740 to 0,9421 |  |
|  | TDP43 | -0,8527 | --0,9969 to 0,6009 |  |
|  | Aβ | -0,2152 | -0,9747 to 0,9404 |  |
|  | BACE | -0,4237 | -0,9841 to 0,9066 |  |
|  | AICD | -0,5405 | -0,9882 to 0,8754 |  |
|  |  |  |  |  |
|  |  |  |  |  |
| **NICD vs** | APP | 0,8939 | -0,4774 to 0,9978 |  |
|  | BACE | 0,5767 | -0,8625 to 0,9894 |  |
|  | Aβ | 0,07654 | -0,9548 to 0,9666 |  |
|  | TDP43 | 0,01228 | -0,9602 to 0,9620 |  |
|  | Fe65 | -0,9025 | -0,9980 to 0,4424 |  |
|  | ADAM10 | 0,0648 | -0,9559 to 0,9658 |  |
|  | ADAM17 | 0,1929 | -0,9431 to 0,9735 |  |
|  | AICD | 0,8227 | -0,6615 to 0,9962 |  |
|  |  |  |  |  |
|  |  |  |  |  |
