# Supplementary material for: Notch Signalling in the Hippocampus of Patients With Motor Neuron Disease
Source: Front Neurosci. 2019 Apr 5;13:302. doi: 10.3389/fnins.2019.00302 (PMC6460507; doi:10.3389/fnins.2019.00302)
Supplement: Supplementary file 3 [file Table_3.docx]

*Supplementary Table 3****.***

*Clinical correlations*

|  | | **ALS1** | **ALS2** | **ALS3** | **ALS/FTD1** | **ALS5** | **ALS6** | **ALS/FTD2** | **ALS8** | **ALS9** | **ALS10** | **ALS11** | **ALS12** |
| --- | --- | --- | --- | --- | --- | --- | --- | --- | --- | --- | --- | --- | --- |
| **SEX** | | M | F | M | M | M | M | F | F | F | M | F | M |
| **AGE AT DEATH** | | 37 | 74 | 70 | 67 | 60 | 46 | 67 | 86 | 81 | 87 | 79 | 51 |
| **CAUSE OF DEATH** | | ARF | ARF | ARF | ARF | ARF | ARF | CRA | ARF | ARF | ARF | STE | ARF |
| **COGNITIVE IMPAIRMENT** | | No | No | No | FTD 4 yr before ALS | No | No | PPA 2 yr before ALS | No | No | No | No | No |
| **FAMILY HISTORY OF COGNITIVE IMPAIRMENT** | | No | Unspecified dementia | No | No | No | No | No | No | No | No | No | No |
| **RELATED ALS GENETIC** | | YES SOD1 | YES SOD1 | No | No | No | No | No | No | No | YES SOD1 | YES TARDBP | No |
| **ALS RELATED PARAMETERS** | **AGE AT DIAGNOSIS** | 33 | 73 | 70 | 62 | 59 | 45 | 65 | 86 | 81 | 83 | 78 | 47 |
|  | **SYNTOMS TO DIAGNOSIS (MONTHS)** | 6 | 13 | 2 | 3 | 11 | 6 | 6 | 2 | 9 | 8 | 12 | 12 |
|  | **SYNTOMS TO DEATH (MONTHS)** | 57 | 27 | 8 | 10 | 18 | 17 | 6 | 3 | 12 | 26 | 24 | 48 |
|  | **ALS ONSET** | Spinal | Bulbar | Spinal | Bulbar | Spinal | Bulbar | Bulbar | Bulbar | Bulbar | Spinal | Bulbar | Spinal |
|  | **RILUZOLE** | Yes | Yes | Yes | Yes | Yes | Yes | No | No | Yes | Yes | Yes | Yes |
|  | **RESPIRATORY ASSISTANT** | Yes | Yes | Yes | No | Yes | Yes | No | No | Yes | Yes | No | Yes |
|  | **GASTROSTOMY** | Yes | Yes | No | No | No | Yes | Yes | No | Yes | No | No | No |

Legend: ALS: Amyotrophic Lateral Sclerosis; M: Male; F: Female; ARF: Acute Respiratory Failure; CRA: Cardiorespiratory Arrest; FTD: Frontotemporal Dementia; Stroke: STE; PPA: Primary Progressive Aphasia
