# Supplementary figures and images for: Notch Signalling in the Hippocampus of Patients With Motor Neuron Disease
Source: Front Neurosci. 2019 Apr 5;13:302. doi: 10.3389/fnins.2019.00302 (PMC6460507; doi:10.3389/fnins.2019.00302)

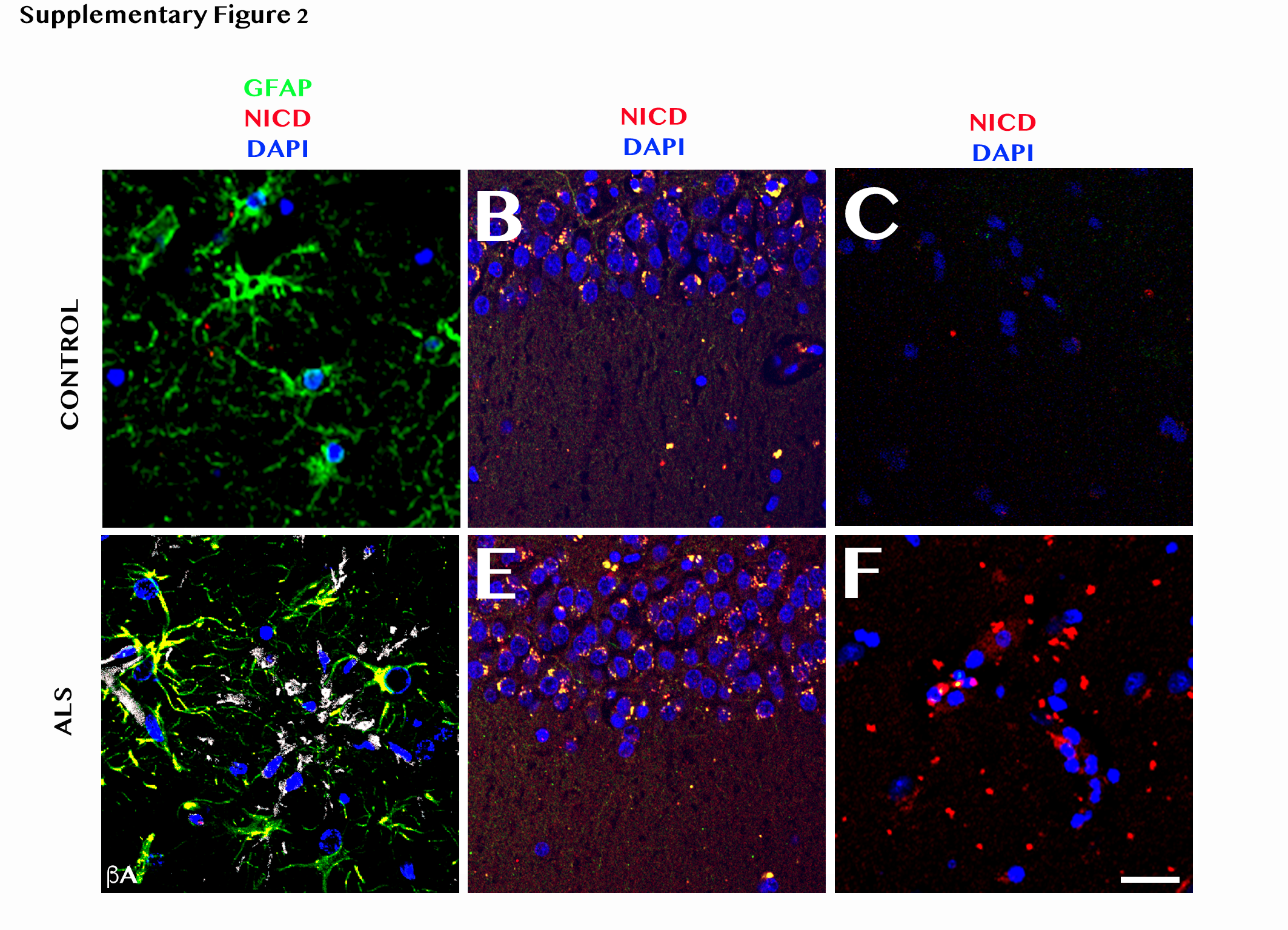

Supplement: Supplementary file 4 [file Image_1.TIF]

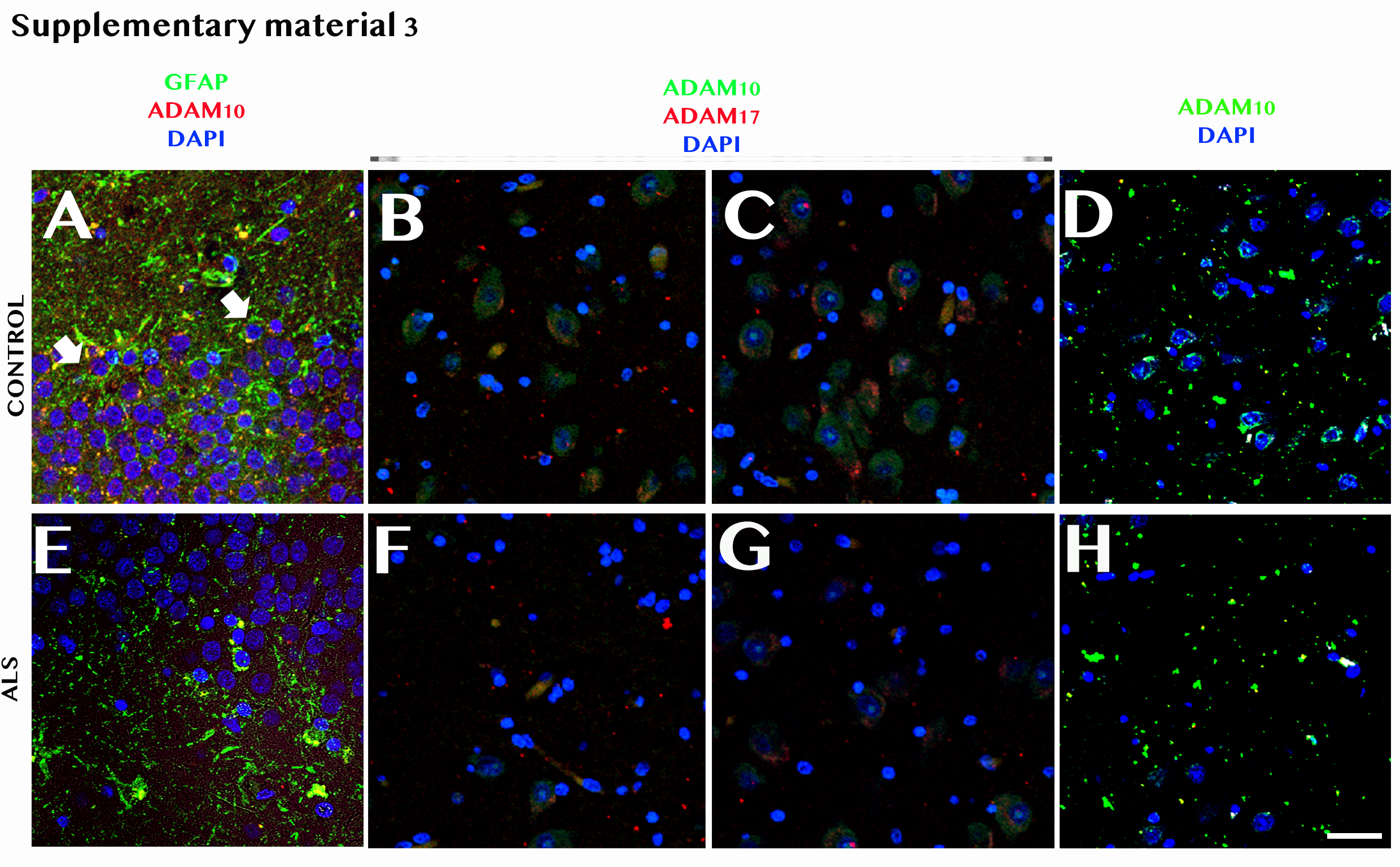

Supplement: Supplementary file 5 [file Image_2.TIF]

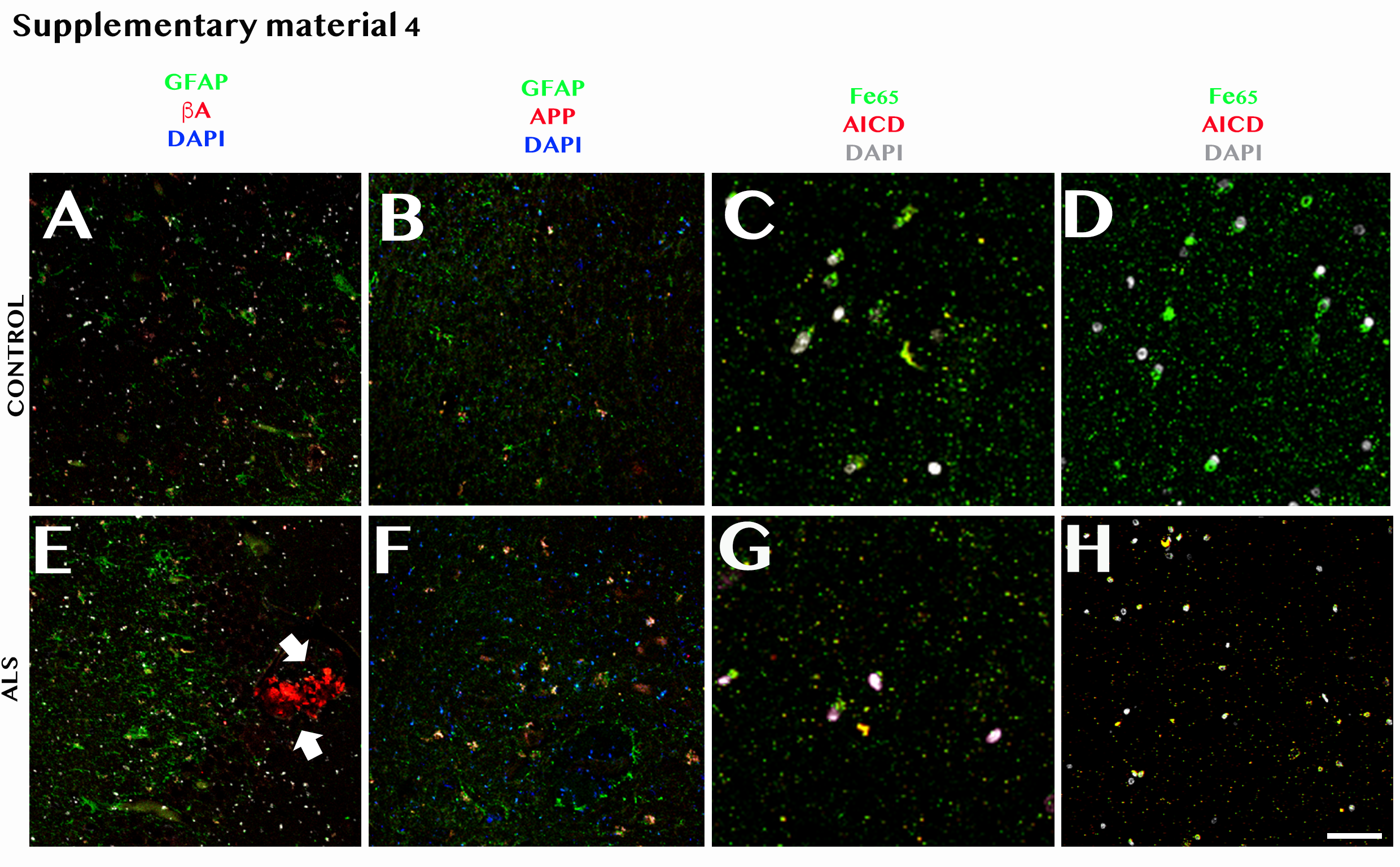

Supplement: Supplementary file 6 [file Image_3.TIF]

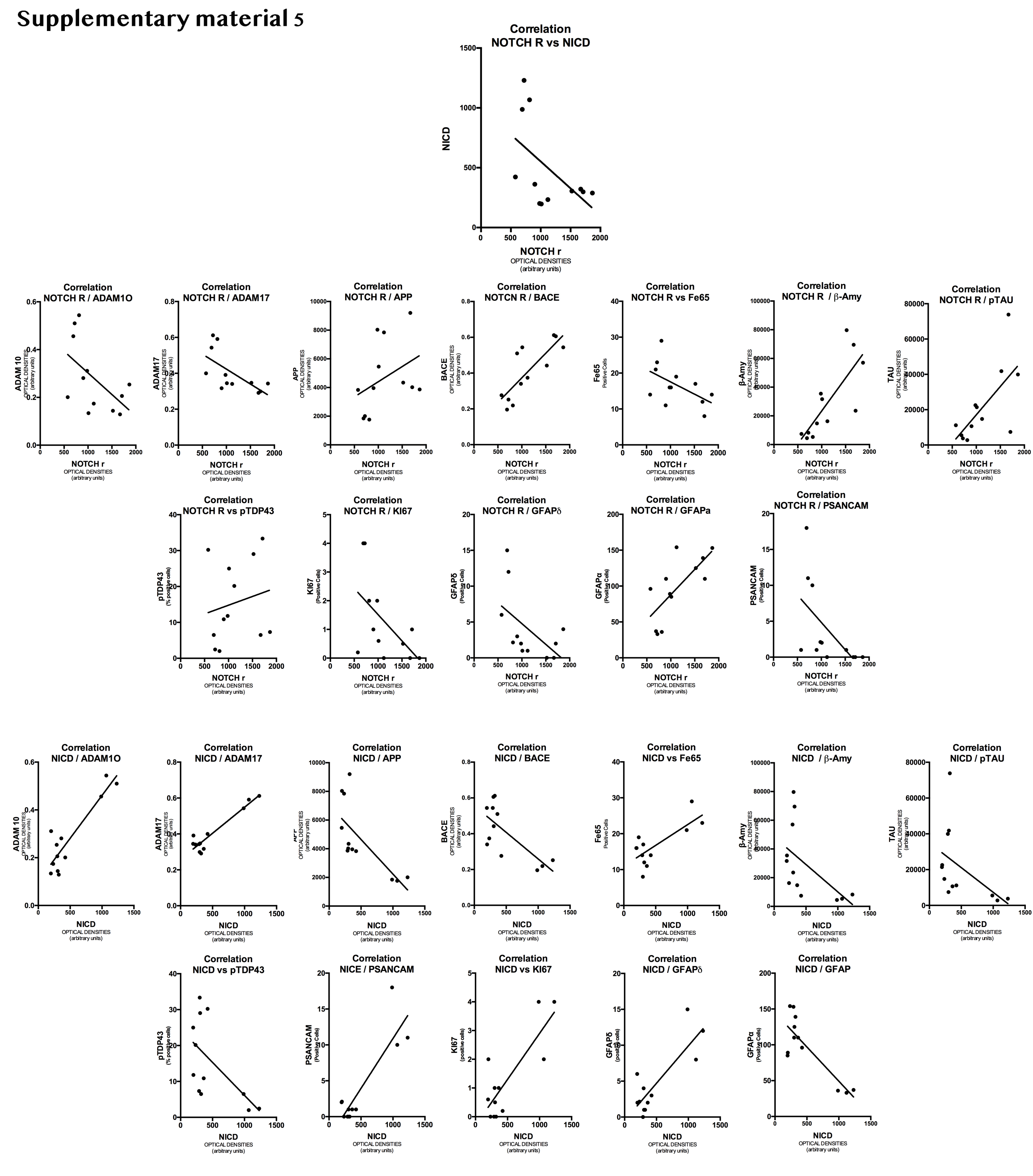

Supplement: Supplementary file 7 [file Image_4.TIF]
