# Supplementary material for: Notch Signalling in the Hippocampus of Patients With Motor Neuron Disease
Source: Front Neurosci. 2019 Apr 5;13:302. doi: 10.3389/fnins.2019.00302 (PMC6460507; doi:10.3389/fnins.2019.00302)
Supplement: Supplementary file 8 [file Data_Sheet_1.docx]

*SUPPLEMENTARY MATERIAL*

*Supplementary material 1.*

*Procedure for autopsy and list of antibodies used.* A) Preparation and storage of biological material. In accordance with our hospital’s protocol and the relevant Spanish legislation, autopsies were performed 2 to 6 hours after patient death. The cranial cavity was opened and the upper end of the spinal cord severed at the level of the foramen magnum. A cut was made along the midline of the corpus callosum to separate the 2 hemispheres. Tissue samples were fixed in 10% buffered formalin with 0.1M PBS (pH 7.35). The hemisphere used for histological and immunohistochemical studies was placed on a flat surface and sliced into 1-cm coronal sections (anterior to posterior). All brains were weighed and macroscopic and microscopic morphology was studied. The microscopic study involved haematoxylin-eosin, Nissl, and thioflavin S staining for all patients; the latter stain was also used for controls. Whenever the pathologist judged it necessary, additional staining techniques, such as Congo red stain and silver staining, were used to detect such macroscopic and microscopic alterations as atrophy, senile plaques, eosinophilia, or neuronal loss. B) Study of the hippocampus. Following the protocol established by our hospital’s anatomical pathology department, hippocampal tissue samples, which included at least CA1, CA2, CA3, and the dentate gyrus, were processed and embedded in paraffin. Samples were cut into 6-µm sections using a Leica microtome to enable study of the cytoarchitecture of the hippocampus. Braak and Braak staging was used to evaluate pathological findings. C) Immunohistochemical study. The immunohistochemical study included the markers Aβ, APP, and phospho-tau. To avoid overlapping, we used 5 slides with hippocampal sections (CA1, CA2, CA3, and dentate gyrus) separated by a distance of at least 200 µm. To deparaffinise tissue samples, we washed them with 0.1M PBS and incubated them at 96°C for 30 minutes in 10mM sodium citrate buffer (pH 6) for epitope retrieval. The samples allocated for immunostaining with Aβ were incubated for 20 minutes in formic acid (v/v). We then added blocking solution to tissue samples (PBS, 0.2% Triton X-100, 10% normal goat serum) for one hour. Tissues were incubated for 24 hours at 4ºC in PBS plus the corresponding primary antibody; they were subsequently washed with PBS and incubated for 2 hours at room temperature with the secondary antibody plus the corresponding Alexa Fluor dye. FluorSave mounting medium (Calbiochem) and an Olympus FV1000 confocal microscope were used for immunofluorescence. The quantitative study included the analysis of 10 different fields for each marker. Results are expressed as the mean of 10 measurements. The ImageJ software (version 1.46r), developed by the National Institutes of Health, was used to measure optical density. Inclusions were quantified based on the number of stained inclusions in neuron cytoplasms divided by the mean number of neurons per field (percentage of cells per mm^2^). D) List of antibodies used.

| **Primary antibodies** | | | | | | |
| --- | --- | --- | --- | --- | --- | --- |
| **Antibody** | **Host** | **Dilution** | **Source/catalogue** | | **Description** | |
| **APP** | Rabbit | 1:200 | Sigma-Aldrich, A8717 | | Marker of amyloid precursor protein (APP) | |
| **APP** | Rabbit | 1:100 | Novus NBP1-76910 | | Marker of APP | |
| **Aβ** | Mouse | 1:1000 | STABvida 123.001.002 | | Marker of amyloid-β protein. The affinity of this antibody is high for the C-terminal epitope of amyloid β 1-42 and 1-40. | |
| **AICD** | Rabbit | 1:200 | BioLegend, 811901 | | Marker of APP intracellular domain (AICD), a peptide fragment that results from intramembrane proteolysis of APP | |
| **NOTCH** | Rabbit | 1:200 | Abcam, ab52627 | | Marker of the receptor for membrane-bound ligands Jagged1, Jagged2, and Delta1, regulating cell fate determination | |
| **NICD** | Rabbit | 1:200 | Abcam, ab8925 | | Marker of NOTCH intracellular domain (NICD) | |
| **Fe65** | Mouse | 1:100 | Novus H00000322-B01P | | Actin-binding protein that enhances membrane ruffling and Rac activation. Enhances the actin-bundling activity of LCP1. May play a role in macrophage activation and function | |
| **Phospo-tau** (pSer396) | Rabbit | 1:500 | Sigma-Aldrich  SAB4504557 | | Promotes microtubule assembly and stability, and might be involved in establishing and maintaining neuronal polarity | |
| **ADAM10** | Mouse | 1:500 | Abcam ab59482 | | Responsible for the proteolytic release of several other cell-surface proteins, including heparin-binding epidermal growth-like factor, ephrin-A2, and for constitutive and regulated α-secretase cleavage of APP | |
| **ADAM17** | Rabbit | 1:500 | Abcam ab2051 | | Responsible for the proteolytic release of several other cell-surface proteins, including p75 TNF-receptor, interleukin 1 receptor type II, p55 TNF-receptor, transforming growth factor-α, L-selectin, growth hormone receptor, MUC1, and APP. Also involved in the activation of Notch pathway | |
| **BACE1** | Rabbit | 1:200 | Abcam ab10716 | | Responsible for the proteolytic processing of APP. Cleaves at the N-terminus of the Aβ peptide sequence, between residues 671 and 672 of APP, leading to the generation and extracellular release of beta-cleaved soluble APP, and a corresponding cell-associated C-terminal fragment which is later released by gamma-secretase | |
| **IBA1** | Goat | 1:300 | Novus NB100-1028 | | Actin-binding protein that enhances membrane ruffling and Rac activation. Enhances the actin-bundling activity of LCP1. Binds calcium. Plays a role in Rac signalling and in phagocytosis. May play a role in macrophage activation and function | |
| **NeuN** | Mouse | 1:500 | Millipore MAB377 | | NeuN antibody (NEUronal Nuclei; clone A60) specifically recognises the DNA-binding, neuron-specific protein NeuN, which is present in most CNS and PNS neuronal cell types of all vertebrates tested. | |
| **TDP43** | Rabbit | 1:500 | Abcam ab109535 | | DNA- and RNA-binding protein which regulates transcription and splicing. Involved in the regulation of CFTR splicing | |
| **PCNA** | Rat | 1:100 | Abcam A29 | | Proliferation marker | |
| **Ki-67** | Mouse | 1:100 | Dako/ M7240 | | Cell proliferation marker (all phases of the cell cycle except G0) | |
| **PSA-NCAM** | Mouse | 1:400 | AbCys/ AbC0019  Clone 355 | | Marker of migrating neuroblasts | |
| **DCX** | Goat | 1:500 | Santa Cruz SC-8066 | | Neuroblast marker | |
| **Nestin** | Mouse | 1:200 | ABCAM AB22035 | | Neural immature progenitors, glia | |
| **GFAPδ** | Rabbit | 1:500 | Millipore/ AB9598 | | Exclusively marks the δ isoform of astrocytes. Neural progenitor cells. Does not recognise GFAPα astrocytes | |
| **GFAPα** | Rabbit | 1:600 | Dako/ Z0334 | | Astrocyte marker | |
| **GFAPα** | Mouse | 1:600 | Sigma/ C9205  Clone G-A-5Cy3 | | Astrocyte marker | |
| **Secondary antibodies** | | | | | | |
| **Antibody** | | | | **Host** | **Dilution** | **Source/catalogue** |
| Alexa Fluor 555 anti Mouse IgG | | | | Goat | 1:500 | Invitrogen/A21424 |
| Alexa Fluor 555 anti Mouse IgM | | | | Goat | 1:500 | Invitrogen/A21426 |
| Alexa Fluor 488 anti Mouse IgG | | | | Goat | 1:500 | Invitrogen/A11029 |
| Alexa Fluor 647 anti Rabbit IgG | | | | Goat | 1:500 | Invitrogen/A21245 |
| Alexa Fluor 488 anti Rat IgG | | | | Goat | 1:500 | ABCAM/ab150165 |
| Alexa Fluor 488 anti Goat IgG | | | | Donkey | 1:500 | Invitrogen/A11055 |
