# Supplementary material for: Notch Signalling in the Hippocampus of Patients With Motor Neuron Disease
Source: Front Neurosci. 2019 Apr 5;13:302. doi: 10.3389/fnins.2019.00302 (PMC6460507; doi:10.3389/fnins.2019.00302)
Supplement: Supplementary file 9 [file Data_Sheet_2.docx]

**Figure legends**

*Supplementary material 2-5*

*Supplementary material 2. Other images of NICD expression.* Confocal microscopy images taken from patients with ALS and controls, showing different hippocampal areas expressing NICD. A-B and D-E: granular layer and subgranular zone. C, F: CA4. Images A-C are from controls, and the remaining images are from patients with ALS. Scale bar: 50 µm.

*Supplementary material 3. α-Secretases, images for ADAM10 and ADAM17.* Confocal microscopy images taken from patients with ALS and controls, showing different hippocampal areas coexpressing GFAP and ADAM10 (A, Arrows). A, E: granular layer and subgranular zone. B, C and F, G: CA4. D,H: CA1. Images A-D are from controls, and the remaining images are from patients with ALS. Scale bar: 50 µm.

*Supplementary material 4. Amyloid cascade: APP, Aβ, AICD, and Fe65 expression*. Confocal microscopy images taken from patients with ALS and controls, showing different hippocampal areas expressing different cellular markers. Images A-D are from controls, and the remaining images are from patients with ALS. Scale bar: 75 µm.

*Supplementary material 5. Immunohistochemical markers.* Graphs of the correlations between the immunohistochemical markers used and Notch1 and NICD. Notch1 shows a positive correlation (r > 0.50) with Aβ, BACE, and tau and an inverse, negative correlation (r < –0.50) with the markers of neurogenesis (Ki67, GFAPδ, PSA-NCAM), Fe65, ADAM10, and ADAM17. NICD is positively correlated with Fe65, ADAM10, and ADAM17 (r > 0.50) and negatively correlated with Aβ, BACE, and tau (r < –0.50).

*Supplementary Table 1.* **Describe the correlations between molecular markers of NOTCH pathway and the correlation with NOTCH and adult neurogenesis in patients.**

*Supplementary Table 2.* **Describe the correlations between molecular markers of NOTCH pathway and the correlation with NOTCH and adult neurogenesis in controls.**

*Supplementary Table 3.* **Describe the clinical features of patients**
